# Clinical outcome of robot-assisted residual mass resection in metastatic nonseminomatous germ cell tumor

**DOI:** 10.1007/s00345-020-03437-z

**Published:** 2020-09-21

**Authors:** Joost M. Blok, Henk G. van der Poel, J. Martijn Kerst, Axel Bex, Oscar R. Brouwer, J. L. H. Ruud Bosch, Simon Horenblas, Richard P. Meijer

**Affiliations:** 1 Department of Oncological Urology, University Medical Center Utrecht, Utrecht, The Netherlands; 2 Department of Urology, The Netherlands Cancer Institute, Utrecht, The Netherlands; 3 Department of Medical Oncology, The Netherlands Cancer Institute, Amsterdam, The Netherlands

**Keywords:** Nonseminomatous germ cell tumor, Retroperitoneal lymph node dissection, Robot-assisted retroperitoneal lymph node dissection, Robotic surgery, Testicular cancer, Testicular germ cell tumor

## Abstract

**Purpose:**

To evaluate the outcome of robot-assisted residual mass resection (RA-RMR) in nonseminomatous germ cell tumor (NSGCT) patients with residual tumor following chemotherapy.

**Patients and methods:**

Retrospective medical chart analysis of all patients with NSGCT undergoing RA-RMR at two tertiary referral centers between January 2007 and April 2019. Patients were considered for RA-RMR in case of a residual tumor between 10 and 50 mm at cross-sectional computed tomography (CT) imaging located ventrally or laterally from the aorta or vena cava, with normalized tumor markers following completion of chemotherapy, and no history of retroperitoneal surgery.

**Results:**

A total of 45 patients were included in the analysis. The Royal Marsden stage before chemotherapy was IIA in 13 (28.9%), IIB in 16 (35.6%), IIC in 3 (6.7%) and IV in 13 patients (28.9%). The median residual tumor size was 1.9 cm (interquartile range [IQR] 1.4–2.8; range 1.0–5.0). Five procedures (11.1%) were converted to an open procedure due to a vascular injury (*n* = 2), technical difficulty (*n* = 2) or tumor debris leakage (*n* = 1). A postoperative adverse event occurred in two patients (4.4%). Histopathology showed teratoma, necrosis and viable cancer in 29 (64.4%), 14 (31.1%), and two patients (4.4%), respectively. After a median follow-up of 41 months (IQR 22–70), one patient (2.2%) relapsed in the retroperitoneum. The one- and 2-year recurrence-free survival rate was 98%.

**Conclusion:**

RA-RMR is an appropriate treatment option in selected patients, potentially providing excellent cure rates with minimal morbidity. Long-term outcome data are needed to further support this strategy and determine inclusion and exclusion criteria.

**Electronic supplementary material:**

The online version of this article (10.1007/s00345-020-03437-z) contains supplementary material, which is available to authorized users.

## Introduction

Approximately one-third of patients who undergo cisplatin-based combination chemotherapy for disseminated nonseminomatous germ cell tumor (NSGCT) have significant residual retroperitoneal disease [1, 2]. Histopathological analysis after postchemotherapy retroperitoneal lymph node dissection (PC-RPLND) shows fibrosis or necrosis in 40–50%, teratoma in 30–40%, and viable cancer in 10–20% of cases [3, 4]. Since there are currently no validated methods to reliably predict the histology of a residual mass, PC-RPLND remains important in all patients with significant residual disease in NSGCT [5].

There is a debate concerning the anatomical extent of PC-RPLND. Historically, bilateral template-based retroperitoneal lymph node dissection was the standard approach in all patients undergoing PC-RPLND [5]. Heidenreich et al. showed that a modified template decreases morbidity and does not compromise oncological outcome in selected patients [4]. Although a template-based procedure is the standard approach, several centers consider residual mass resection as oncologically equivalent [6, 7].

More recently, the minimally invasive approach is gaining recognition in the post-chemotherapy setting. Two large series have shown excellent oncological outcomes after laparoscopic PC-RPLND [8, 9], but high volume series on robot-assisted PC-RPLND (RA-PC-RPLND) are still lacking [10]. In the largest series to date, none of the 30 patients undergoing RA-PC-RPLND had retroperitoneal relapse [11]. These promising initial results and the continuous evolvement of surgical techniques and technology suggest that robotic surgery may replace open PC-RPLND in selected patients. On the condition that oncological safety is warranted, this may provide significant benefit to patients. After all, the morbidity of open PC-RPLND is high, while histopathology of the retroperitoneal specimen shows fibrosis or necrosis in a large proportion of patients [3, 4, 12, 13].

Current reports on minimally invasive PC-RPLND mainly concern template-based surgery. We hypothesized that, in selected patients, oncological control can be achieved by robot-assisted residual mass resection (RA-RMR). In this study, we retrospectively evaluated the results of this approach in two tertiary referral centers.

## Patients and methods

### Study design

After institutional review board approval, we retrospectively reviewed the medical charts for all NSGCT patients who underwent post-chemotherapy RA-RMR in two tertiary referral centers between January 2007 and April 2019.

Work-up prior to surgery included abdominopelvic computed tomography (CT) scanning and measurement of serum tumor markers (α-fetoprotein, human chorionic gonadotropin and lactate dehydrogenase). All treatment options were discussed by a multidisciplinary panel consisting of a urological oncologist, medical oncologist, radiologist, radiation oncologist and genitourinary pathologist. Patients were considered for RA-RMR in case of one or two residual tumors between 10 and 50 mm at cross-sectional CT imaging located ventrally or laterally from the aorta or vena cava, with normalized tumor markers following completion of chemotherapy, and no history of retroperitoneal surgery.

### Surgical technique

Patients were positioned in the flank position contralateral of the residual tumor. Exact port placement depended on the location of the residual tumor and the surgeon’s preference. In general, a four-port diamond-shaped method was used. The camera port was placed in the paramedian line 3–4 cm cranial to the umbilicus and three additional ports were placed in the upper quadrant, lower quadrant and flank, including an assistant port. In some cases, a fifth port was placed subcostally in the midline.

**Fig. 1:**
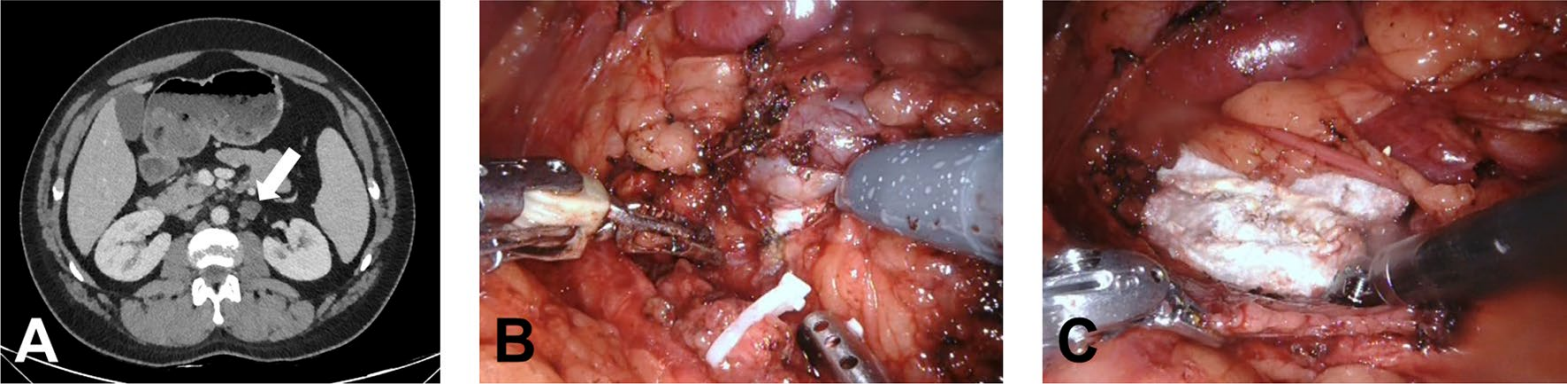
Computerized tomography scan and intraoperative images of patient undergoing RA-RMR. This patient had a residual tumor (short axis 1.3 cm) in the left para-aortal region. Histopathology showed a 3 cm large teratoma. **a** Axial abdominal CT scan after chemotherapy with a residual tumor in the left para-aortal region (arrow). **b** Intra-operative image with the tumor still in situ. **c** Intra-operative image after the tumor has been resected and a Surgicel has been placed in the retroperitoneum. In images (**b**) and (**c**) it is clear that the surrounding nodes and fat are not resected

The surgical resection was not template-based, with the individual extent of the resection adhering to the location of the metastases prior to chemotherapy and the location of the residual tumor (Fig. 1). Any mass in addition to the lesion defined on presurgical CT suspicious for residual tumor that was noticed during surgery was resected as well as lymph nodes in the vicinity and the remnant testicular vessels.

### Follow-up

Follow-up was performed according to current guidelines of the European Society for Medical Oncology. In general, this consisted of monthly clinical examinations and evaluations of serum tumor markers in the first year. After the first year, the frequency of follow-up was gradually reduced every year. Abdominal/thoracic CT scanning was done at least three times (after 6, 12 and 24 months).

## Results

Out of a total of 208 RPLNDs, 67 RA-RMRs were performed. Twenty-two patients were excluded from the current analysis, because (a) they were not treated with chemotherapy prior to surgery (*n* = 15), (b) the operative report was missing (*n* = 2), (c) tumor markers were elevated at time of surgery (n = 2), (d) no NSGCT primary (*n* = 2) or (e) history of prior RPLND (*n* = 1). The remaining 45 patients were included in the analysis (Table 1).

**Table 1:** Patient characteristics and outcome

Number of patients	45
Median age at surgery, *years (IQR)*	29 (23–36)
Primary tumor side, *n *(%)	
Left	32 (71.1)
Right	13 (28.9)
Royal Marsden stage prior to chemo, *n *(%)	
IIA	13 (28.9)
IIB	16 (35.6)
IIC	3 (6.7)
IV	13 (28.9)
IGCCCG prognosis category	
Good	38 (84.4)
Intermediate	6 (13.3)
Poor	1 (2.2)
Cycles of platinum-based chemotherapy, *n *(%)	
3 cycles	24 (53.3)
4 cycles	14 (31.1)
> 4 cycles	1 (2.2)
Unknown	6 (13.3)
Median residual tumor size, *cm (IQR)*	1.9 (1.4–2.8)
Residual tumor location, *n *(%)	
Para-aortic	32 (71.1)
Para-caval	3 (6.7)
Interaortocaval	10 (22.2)
Median operative time, *mins (IQR)*	134 (100–174)
Median intraoperative blood loss, *ml (IQR)*	50 (5–110)
Intraoperative adverse events, *n *(%)	5 (11.1)
Vascular lesion	2 (4.4)
Debris leakage	2 (4.4)
Spleen lesion	1 (2.2)
Conversions to open surgery, *n *(%)	5 (11.1)
Technical difficulty	2 (4.4)
Vascular lesion	2 (4.4)
Debris leakage	1 (2.2)
Postoperative complication, *n *(%)	2 (4.4)
Clavien-Dindo Grade 2	1 (2.2)
Clavien-Dindo Grade 3a	1 (2.2)
Median length of hospitalization, *days (range)*	2 (1–3)
Retroperitoneal histology, *n *(%)	
Necrosis / fibrosis	14 (31.1)
Teratoma	29 (64.4)
Viable cancer	2 (4.4)
Median length of follow-up, *months (IQR)*	41 (22–70)
Relapse, *n *(%)	1 (2.2)
Survival status, *n *(%)	
No evidence of disease	43 (95.6)
Died of other causes	2 (4.4)

*IGCCCG *International Germ Cell Cancer Collaborative Group, *IQR *interquartile range

In 71% of patients, the residual tumor was located in the left para-aortal region. Thirty-eight patients (84.4%) had a solitary tumor on preoperative imaging. Five patients (11.1%) had two nodes and one patient (2.2%) had five nodes. The median tumor size was 1.9 cm (interquartile range [IQR] 1.4–2.8; range 1.0–5.0).

### Adverse events

An intra-operative adverse event was recorded in five patients (11.1%; Table 1). Two vascular injuries occurred: one renal artery injury and one inferior mesenteric artery injury. Both events required conversion to an open procedure. In two patients, debris leaked from the residual tumor, which required conversion to an open procedure in one case. The fifth patient had a splenic injury, most likely due to excessive traction. No bleeding was observed and the injury was coagulated with a bipolar coagulator.

In addition to the three patients who required a conversion due to an intra-operative adverse event, two patients required conversion to an open procedure due to technical difficulties. One patient had a retro-aortic node adhesive to the surrounding tissue which could not be resected during robotic surgery. The node was successfully resected after conversion. The second patient had two residual tumors: one para-aortic node and one node adjacent to the left common iliac vein. The surgeon was able to resect the para-aortic node during the robot-assisted procedure, but resection of the para-iliacal tumor was unsuccessful. After midline laparotomy, the para-iliacal tumor (sized 4 × 3 × 2.5 cm) was successfully resected. Palpation of the para-aortal region revealed two additional small nodes which were resected and were confirmed to contain teratoma at histopathology.

Two patients (4%) had a postoperative adverse event Clavien-Dindo grade ≥ 2. One patient was readmitted 22 days after surgery for a 9 cm large lymphocele with urinary tract obstruction and secondary pyelonephritis. He was treated with intravenous antibiotics (grade 2 complication). The second patient too was readmitted with a lymphocele six days after surgery (four days after hospital discharge). A drain was placed and a medium-chain triglyceride diet was prescribed (grade 3a).

### Histology

The median number of resected nodes was three (IQR 1–6). The retroperitoneal specimen showed teratoma, necrosis and viable cancer in 29 (64%), 14 (31%), and 2 patients (4%), respectively. Since the amount of viable cancer was < 10% in both patients, they were not treated with additional chemotherapy.

### Follow-up

The median follow-up of the entire cohort was 41 months (IQR 22–70). Follow-up was shorter than 1 year in three patients, who preferred to have their follow-up visits at the referring hospital. Based on only one patient with disease progression, the 1- and 2-year relapse-free survival rates were 98%.

One patient had disease progression with elevated tumor markers. The CT-scan of this patient, prior to RA-RMR, showed a 1.5 cm large residual tumor cranial to the left renal vessels. The CT-scan 3 months after surgery showed a 2.9 cm large para-aortic node at the same location, which suggests that the residual tumor was overlooked during surgery and not adequately resected. In addition, a 2.9 cm large node in the interaortocaval region was found. A CT-scan prior to chemotherapy had shown minimal growth of small interaortocaval nodes, but there was no residual tumor visible in the interaortocaval region after completion of chemotherapy. Subsequent treatment with salvage chemotherapy and open RPLND was successful and he had no evidence of disease after 83 months of follow-up.

None of the patients died of disease but two patients died of other causes. One patient died 11 months after surgery due to acute leukemia. Another patient died of renal cell carcinoma, more than 4 years after surgery.

## Discussion

We report the perioperative and oncologic outcomes in a series of 45 selected NSGCT patients undergoing RA-RMR. Two patients (4.4%) had a postoperative complication Clavien-Dindo grade ≥ 2 with short admission time and one patient (2.2%) had disease progression in the retroperitoneum. After a median follow-up of more than 3 years, none of the patients had evidence of disease.

Patients with a residual tumor after chemotherapy for disseminated NSGCT form a unique group of cancer patients. They are relatively young and long-term survival is expected in most cases [14]. Although surgical resection of viable cancer is important, histopathological examination of the retroperitoneal specimen shows necrosis in most patients [4, 15]. In addition, the presentation of patients with testicular cancer is changing. The proportion of patients initially presenting with low-stage disease is increasing and systemic chemotherapy is applied more often in patients with low-volume retroperitoneal metastases [16, 17]. Non-cancer histology is especially common in patients with a small residual lesion [15]. These aspects highlight the increasing importance of the reduction of treatment-associated morbidity and shift the focus of testicular germ cell tumor (TGCT) treatment to a more patient-tailored approach.

Maintaining oncological efficacy is an important prerequisite for the adoption of a minimally invasive approach and several series on minimally invasive PC-RPLND have shown promising results (Supplementary Table 1) [7–9, 11, 18–20]. Steiner et al. reported on 100 patients that were treated with a unilateral (*n* = 71) or bilateral (*n* = 29) laparoscopic template dissection [9]. Patient characteristics were relatively favorable, since the largest tumor diameter was < 1 cm in 51/100 patients. Only one relapse (outside the surgical field) was observed after a mean follow-up of > 5 years.

Another key study is a series of 67 patients by Nicolai et al. [8]. Contrary to the series by Steiner et al. only patients with a clinically significant residual tumor (1–5 cm) were eligible. Although the median follow-up was only 21 months, none of the patients relapsed. These promising findings are supported by a recent systematic review, which found a weighted average retroperitoneal relapse rate of minimally invasive PC-RPLND of only 1.7% [10].

For RA-PC-RPLND specifically, the data on oncological safety are not yet mature enough to draw firm conclusions [10, 21]. In the largest cohort to date, Li et al. retrospectively analyzed the outcome of 30 patients undergoing template-based RA-PC-RPLND and compared this with a cohort of patients treated with open resection [11]. None of the patients in the robot-assisted group relapsed in the retroperitoneum.

Several studies have shown that completeness of the residual tumor resection is an important factor in oncological outcome [22, 23]. Fléchon et al. reported the results of 151 patients treated with open PC-RPLND between 1992 and 2002 with the aim to determine whether conformity to the recommendations of the Memorial Sloan Kettering Cancer Center (MSKCC) and completeness of the resection are associated with oncological outcome [22]. Of the 70 patients with a complete resection according to the MSKCC recommendations, only two patients (2.9%) had a retroperitoneal relapse. In the group of 58 patients with a complete resection, but not according to the MSKCC recommendations, three patients (5.2%) had a retroperitoneal relapse. If patients with an incomplete resection are also considered, thirteen out of 81 patients with a compliant but incomplete resection or with a non-compliant complete or incomplete resection relapsed in the retroperitoneum (16%). This corresponded to an event-free survival probability at 10 years of 72%, compared to 85% for patients with compliant and complete resection. It should be noted that the initial tumor was ≥ 5 cm in fourteen out of fifteen patients with retroperitoneal relapse. In our series, none of the patients had a residual tumor > 5 cm. Nevertheless, this study shows that conformity to the guidelines and completeness of the resection might have an effect on oncological outcome [22].

In another large series of patients undergoing open RMR, seven out of 97 patients with macroscopically complete resection (7%) suffered from retroperitoneal relapse [6]. As with the study by Fléchon et al., patient characteristics were relatively worse compared to our cohort, since more than half of patients had a residual tumor > 4 cm. Both studies show that RMR may not be an appropriate approach in patients with large residual tumors.

In a randomized comparison of chemotherapeutic regimens, complete resection was mandatory without stating the extent of the template [24]. Four out of 100 patients with normalized tumor markers and nonviable histology of residual tumor (4%) relapsed. In the group with normalized tumor markers and viable histology of residual tumor, four out of eleven patients (36%) relapsed. In our series, RA-RMR was only considered in those cases, where complete resection of the residual lesion was considered possible.

The literature on minimally invasive RMR is scarce. Öztürk et al. described the results of laparoscopic RMR in a series of 89 patients treated between 2005 and 2015 [7]. Eight patients (9%) of the entire cohort relapsed, or four out of 75 procedures that were completed laparoscopically (5%). This relatively high relapse rate may be explained by the substantial number of patients with vital cancer in the retroperitoneum: 16% versus 4.4% in our cohort. In addition, three of the relapsed patients had interaortocaval tumor spread and two had contralateral tumor spread, which would have justified a bilateral dissection according to the Heidenreich criteria [4]. In a series of 12 patients undergoing RA-PC-RPLND by Kamel et al., three patients were treated with RA-RMR [20]. None relapsed after a follow-up of 5, 22, and 30 months.

In our cohort, one patient had tumor progression. This was partly due to an incomplete resection, but also due to a retroperitoneal relapse in the interaortocaval region outside the surgical field. If this patient would have been treated with a template-based approach, this probably would have been a left-sided modified template, since interaortocaval dissemination is highly unusual in patients with a left-sided primary tumor [25] and the para-aortic residual tumor was only 1.5 cm. This approach would not have prevented the interaortocaval relapse.

An important benefit of minimally invasive surgery is the improved perioperative outcome, compared to open surgery [8, 9, 26–28]. Robot-assisted surgery has additional benefits such as 360° movement of instruments, ability of three dimensional vision, better surgeon ergonomics, and accuracy and stability in confined spaces [21, 29]. The only major complication in our series was a lymphocele requiring drainage. This is in contrast with several population-based studies on open RPLND, which have reported average complication rates of ~ 25% [30, 31].

The duration of follow-up in the present study is relatively long, but it is not long enough to safely rule out any future retroperitoneal relapses. Although rare, relapse after complete remission following chemotherapy is possible even beyond 5 years of follow-up [2, 32, 33].

Several studies have shown that patient outcome after complex cancer surgery is correlated with hospital volume [10, 31, 34]. In patients with advanced TGCT, higher hospital volume is associated with improved survival outcomes [35] and high volume hospitals have fewer post-operative complications and more routine home discharges after RPLND [31]. Therefore, patients with advanced TGCT should be managed at high volume expert centers.

Our study is subject to certain limitations. The major limitation is its retrospective design. There were no strict predefined inclusion and exclusion criteria, which may have introduced bias in patient selection. In addition, postoperative antegrade ejaculation was not routinely recorded, which is an important aspect of retroperitoneal surgery.

It is unlikely that all open procedures will be replaced by a minimally invasive approach. In case of a large residual tumor, infiltration or encasement of the large vessels, retro-aortic or retro-caval tumor location, or if an additional surgical intervention (e.g., nephrectomy) is indicated, open surgery may still be the preferred approach. At the same time, the criteria for a minimally invasive procedure are dynamic instead of fixed. Surgical techniques, surgeon experience and technological innovations keep evolving, which will expand the indication of the minimally invasive approach. For example, the feasibility of a bilateral template dissection without patient repositioning has already been shown [36] and Aufderklamm et al. have reported laparoscopic PC-RPLND with vascular reconstruction in patients with a residual tumor infiltrating the large vessels [37]. Rapidly developing robot-assisted techniques will expand the indication even further.

RMR has been the standard management for post-chemotherapy resection at our institute since 1979. Not all residual tumor patients are suitable for RMR instead of template dissection. According to the Heidenreich criteria, patients with contralateral tumor spread, residual tumor > 5 cm or interaortocaval location should undergo a bilateral instead of unilateral template dissection [4]. Thus, they are also not eligible for RMR.

In addition, patients with multiple enlarged nodes post-chemotherapy may have an increased risk of microscopic residual teratoma or vital cancer elsewhere in the retroperitoneum and are preferably treated with a template based procedure. It is also conceivable that the extent of the tumor prior to chemotherapy plays an important role. Pre-chemotherapy retroperitoneal nodal size and presence of visceral metastases are associated with relapse after PC-RPLND [3]. Patients with supradiaphragmatic node involvement or multiple tumors prior to chemotherapy may also have an increased risk of residual tumor beyond what is visible on post-chemotherapy CT-scans.

In summary, RA-RMR may be an appropriate treatment option in patients with a single tumor in the primary landing zone which has not extended beyond 5 cm in diameter since initial diagnosis. However, further studies are necessary to establish the inclusion and exclusion criteria for a more limited dissection.

RA-RMR encompasses two developments: RMR instead of template resection and robot-assisted surgery instead of open surgery. It is important to bear in mind that there are currently no high-volume long-term data on either development. Since RA-RMR is a more limited approach than conventional PC-RPLND, sufficient follow-up is especially important. At the very least, patients should be considered as if they have been treated with a template-based PC-RPLND and thus followed for 5 years. However, it could be the case that patients need to be followed for a longer period of time (e.g., up to 10 years), because they underwent a more limited resection. This is an important topic for further research.

## Conclusion

RA-RMR may be an appropriate treatment option in selected patients, potentially providing excellent cure rates with minimal morbidity at intermediate follow-up. Long-term outcome data are needed to further support this strategy and determine inclusion and exclusion criteria.

## Electronic supplementary material

Below is the link to the electronic supplementary material.

Supplementary file1 (DOCX 22 kb)

## Abbreviations

CT: Computerized tomography
IGCCCG: International Germ Cell Cancer Collaborative Group
IQR: Interquartile range
MSKCC: Memorial Sloan Kettering Cancer Center
NSGCT: Nonseminomatous germ cell tumor
TGCT: Testicular germ cell tumor
PC-RPLND: Postchemotherapy retroperitoneal lymph node dissection
RA-PC-RPLND: Robot-assisted postchemotherapy retroperitoneal lymph node dissection
RA-RMR: Robot-assisted residual mass resection
