# Supplementary material for: Clinical outcome of robot-assisted residual mass resection in metastatic nonseminomatous germ cell tumor
Source: World J Urol. 2020 Sep 21;39(6):1969–76. doi: 10.1007/s00345-020-03437-z (PMC8217018; doi:10.1007/s00345-020-03437-z)
Supplement: Supplementary file 1 — Supplementary file1 (DOCX 22 kb) [file 345_2020_3437_MOESM1_ESM.docx]

**Supplementary Table 1. Summary of Outcomes of Minimally Invasive PC-RPLND in Other Reports**

| Study | **Procedure** | **Inclusion criteria** | **No. of patients** | **Residual tumor size** | **Conversion, *n* (%)** | **Postoperative complications, *n* (%)** | **RP histology, *n* (%)** | **RP relapse, *n* (%)** | **Median follow-up** |
| --- | --- | --- | --- | --- | --- | --- | --- | --- | --- |
| Li et al. (2019) [11] | RA | NM | 30^*^ | <1 cm: 7 pts. (26%)  >5 cm: 4 pts. (13%) | 3 (10): inadequate visualization in 2 pts., vascular injury in 1 pt. | 6 (20): wound infection in 2 pts.; delirium tremens; chylous ascites; pneumothorax; colon perforation | Necrosis: 10 (33.3) Teratoma: 15 (50) Vital cancer: 11 (16.7) | None^†^ | 15 months |
| Öztürk et al. (2019) [7] | Lap. | Residual tumor <5 cm and not posterior to large vessels | 89 | Median 2.0 cm (range 0.5-7.0) | 14 (15.7): technical difficulty in 7 pts., patient-related factors in 4 pts., ureter injury, aortic injury, IVC injury | 9 (12) | Necrosis: 33 (37) Teratoma: 42 (47) Vital cancer: 14 (16) | 7 (7.9)^‡^ | 91 months |
| Overs et al. (2018) [18] | RA | NSGCT: residual tumor >1 cm  SGCT: residual tumor >3 cm | 11 | Median 2.0 cm (range 1.25-4.0) | None | 1 (9): chylous ascites | Necrosis: 3 (27)  Teratoma: 8 (73)  Vital cancer: 0 | None | 4 months^\|^ |
| Singh et al. (2017) [19] | RA | Normal markers, single residual tumor at landing zone <6 cm, or multiple tumors over IVC and aorta <5 cm, no organ involvement | 13 | Stage 2a: 7 pts.  Stage 2b: 5 pts.  Stage 2c: 1 pt. | None | 4 (31): chylous ascites | Necrosis: 10 (77)  Teratoma: 3 (23) Vital cancer: 0 | None | 23 months |
| Kamel et al. (2016) [20] | RA | NSGCT: normal markers, residual tumor ≥1 cm  SGCT: residual tumor ≥3 cm / tumor <3 cm PET/CT positive | 12^§^ | ≥5 cm: 10 pts. (83%) | 1 (8): inferior mesenteric artery injury | None | Necrosis: 5 (45.5)  Teratoma: 5 (45.5)  Vital cancer: 1 (9) | None | 31 months |
| Nicolai et al. (2016) [8] | Lap. | No previous RP surgery, unilateral disease since the beginning, residual tumor 1-5 cm, marker normalization, encasement of IVC/aorta <30% of circumference | 67 | Median: 2.7 cm  <1 cm: 1 pt. (1.5%) | 3 (4.5): extensive fibrosis involving IVC/aorta in 2 pts., renal vein injury in 1 pt. | 3 (4.5): retrograde ejaculation; blood transfusion; percutaneous drainage of lymphocele | Necrosis: 14 (20.9) Teratoma: 51 (76.1) Vital cancer: 2 (3.0) | None | 21 months |
| Steiner et al. (2013) [9] | Lap. | No bulky disease | 100 | Mean: 1.4 cm <1 cm: 51 pts. (51%) | 1 (1): vena cava injury | 2 (2): fenestration of lymphocele and peritoneal venous shunt for chylous ascites | Necrosis: 60 (60) Teratoma: 38 (38) Vital cancer: 2 (2) | One patient, outside the surgical field | 59 months |

IVC = inferior vena cava; Lap. = laparoscopic; NM = not mentioned; PET/CT = positron emission tomography / computed tomography; RA = robot-assisted; RP = retroperitoneum

* Including 7 patients (26%) with elevated markers at time of surgery.

† Three patients relapsed at distant sites. Two of these had undergone desperation surgery with viable cancer in the retroperitoneum. The other patient had pulmonary metastases after pulmonary lobectomy and neck dissection in conjunction with PC-RPLND.

‡ Seven out of 8 relapses were in the retroperitoneum (author communication).

§ Including 3 patients with a seminoma primary.

| Long-term follow-up available for 6 patients. None of these patients had recurrence after 24 months of follow-up.
